# Molecular Characterization and Prognostication of Large Cell Neuroendocrine Carcinoma and Large Cell Carcinoma

**DOI:** 10.3389/fonc.2021.664397

**Published:** 2022-01-14

**Authors:** Ying Chen, Xiaoying Cui, Di Wang, Guojie Xia, Minyan Xing, Lei Cheng, Liming Sheng, Xianghui Du

**Affiliations:** ^1^ Department of Radiation Oncology, Zhejiang Key Laboratory of Radiation Oncology, Cancer Hospital of the University of Chinese Academy of Sciences (Zhejiang Cancer Hospital), Institute of Cancer and Basic Medicine (IBMC), Chinese Academy of Sciences, Hangzhou City, China; ^2^ The Second Clinical Medical College, Zhejiang Chinese Medical University, Hangzhou, China; ^3^ Department of Medical, Nanjing Geneseeq Technology Inc, Nanjing, China; ^4^ Department of Oncology, Traditional Chinese Medical Hospital of Huzhou, Huzhou, China; ^5^ Department of Oncology, Haining People’s Hospital, The First Affiliated Hospital of Zhejiang University, Haining, China

**Keywords:** large cell carcinoma, *SMARCA4*, *KEAP1*, *BRAF*, *RB1*

## Abstract

**Purpose:**

Large cell neuroendocrine carcinoma (LCNEC) and classic large cell carcinoma (LCC) are two distinct entities with different histological and biological characteristics. However, the mutational profiles and the clinical behavior of the two subtypes of lung cancer remain to be explored.

**Patients and Methods:**

Pathological diagnoses of all screened patients were finally confirmed by three or four experienced pathologists. Patients with uncertain pathological diagnoses were excluded. Finally, we genetically profiled ten patients with LCNEC and seven with LCC. ALL patients were subjected to next-generation sequencing (NGS) test, which included nine patients sequenced with a 139-gene panel and eight patients with a 425-gene panel. Including only intersected mutations from these two panels, survival analysis was further conducted.

**Results:**

Both LCNEC and LCC showed high prevalence in male patients, with no clear association with smoking history. Potential targetable mutations in *KRAS* and *RET* were detected in the study cohort. However, LCNEC and LCC showed distinct mutational profiles with an enrichment of *RB1*/*TP53* co-mutations in a subset of LCNEC patients. *SMARCA4* and *KEAP1* mutations were exclusively found in LCC patients, and *RICTOR*, *BRAF*, *ROS1* and *TET2* mutations were only detected in LCNEC. LCC patients in the cohort had shorter survival compared to LCNEC patients (p=0.006). Survival analysis revealed an association between *SMARCA4* mutations and poor outcome in the study cohort and in the LCC subset. Mutations in *BRAF* were associated with a trend of increased survival in the study cohort, as well as in the LCNEC subset. Finally, *TET2* mutations were associated with poor outcome in the LCNEC cohort.

**Conclusion:**

LCC and LCNEC were both heterogeneous diseases with limited treatment options. Our study identified potential targetable mutations and prognostic biomarkers that might provide more therapeutic options and improve individualized patient care.

## Introduction

Large-cell carcinoma (LCC) of the lung is an undifferentiated tumor, which lacks of the immunohistochemical and cytological characteristics of adenocarcinoma, squamous cell carcinoma or small-cell carcinoma (1). It is a relatively uncommon lung cancer that is characterized by poor prognosis and limited treatment options. Large cell neuroendocrine carcinoma (LCNEC) was initially classified as a histological variant of LCC. However, LCNEC and LCC differ in their cytological and histological features, as well as in clinical behavior to treatment. It has been reported that LCNEC was associated with worse survival outcome compared to LCC (2). As a consequence, the 2015 World Health Organization (WHO) classification significantly revised the classification of LCC and removed large cell neuroendocrine carcinoma (LCNEC) from the LCC category, establishing LCNEC as a distinct entity (3).

With the advances in next-generation sequencing (NGS), numerous actionable driver genes have emerged from the genetic landscape of different lung cancer subtypes. At the same time, many efforts have been undertaken to clarify the biological relationship between LCNEC, classic LCC and other types of lung cancer (1, 4–6). LCNEC has been segregated into small-cell lung carcinoma (SCLC)-like, characterized by the co-occurrence of *TP53* and *RB1* mutations, and non-small-cell lung carcinoma (NSCLC)-like, characterized by the lack of *TP53*/*RB1* co-mutations and *STK11*/*KEAP1*/*KRAS* mutations (4, 6). On the other hand, classic LCCs are also genetically heterogeneous, displaying profiles characteristics of either adenocarcinoma or squamous cell carcinoma (7). In this study, we aimed to further investigate these two subtypes of lung cancer and identify potential biomarkers for treatment and prognostication of these patients.

## Material and Methods

### Patients and Samples

Pathological diagnoses of all screened patients were finally confirmed by three or four experienced pathologists, and patients with uncertain pathological diagnoses were excluded. Finally, a total of 17 patients, ten patients with LCNEC and seven with LCC, who were treated at Zhejiang Cancer Hospital from March 2017 to April 2020 with sufficient clinical information and follow-up, were included in the study. Samples were profiled using targeted NGS for routine diagnostic or treatment purposes at Nanjing Geneseeq Technology Inc.

### DNA Extraction, Library Preparation and Sequencing

DNA extraction, sequencing library preparation, and targeted capture enrichment were carried out following the methods as previously described with modifications (8). Briefly, Genomic DNA from the white blood cells were extracted using the DNeasy Blood & Tissue Kit (Qiagen) and used as the normal control to remove germline variations. FFPE samples were de-paraffinized with xylene, and genomic DNA was extracted using the QIAamp DNA FFPE Tissue Kit (Qiagen). DNA was quantified by Qubit 3.0 using the dsDNA HS Assay Kit (Life Technologies), and the quality was evaluated by a Nanodrop 2000 (Thermo Fisher).

Libraries were prepared by KAPA Hyper Prep kit (KAPA Biosystems), as previously described (9). Briefly, 1-2 μg of genomic DNA was sheared into ~350 bp fragments using a Covaris M220 instrument. End repair, A-tailing, and adaptor ligation of fragmented DNA were performed using the KAPA Hyper DNA Library Prep kit (Roche Diagnostics), followed by size selection with AgencourtAMPure XP beads (Beckman Coulter). DNA Libraries were then amplified by polymerase chain reaction (PCR) and purified using AgencourtAMPure XP beads.

Customized xGen lockdown probes panel (Integrated DNA Technologies) were used to selectively enrich for 139 or 425 predefined cancer-related genes (Geneseeq panel). Human cot-1 DNA (Life Technologies) and xGen Universal Blocking Oligos (Integrated DNA Technologies) were added as blocking reagents. The capture reaction was performed with Dynabeads M-270 (Life Technologies) and the xGen Lockdown Hybridization and Wash kit (Integrated DNA Technologies). Captured libraries were subjected to PCR amplification with KAPA HiFi HotStartReadyMix (KAPA Biosystems). The purified library was quantified using the KAPA Library Quantification kit (KAPA Biosystems), and its fragment size distribution was analyzed using a Bioanalyzer 2100. Target enriched libraries were sequenced on the HiSeq4000 platform (Illumina).

### Mutation Calling

Sequencing data were demultiplexed by bcl2fastq (v2.19), analyzed by Trimmomatic (10) to remove low-quality (quality<15) or N bases. Then the data were aligned to the hg19 reference human genome with the Burrows-Wheeler Aligner (bwa-mem) (11) and further processed using the Picard suite (available at: https://broadinstitute.github.io/picard/) and the Genome Analysis Toolkit (GATK) (12). SNPs and indels were called by VarScan2 (13) and HaplotypeCaller/UnifiedGenotyper in GATK, with the mutant allele frequency (MAF) cutoff as 0.5%. Common variants were removed using dbSNP and the 1000 Genome project. Germline mutations were filtered out by comparing to patient’s whole blood controls.

### Statistical Analysis

Comparisons of proportion between groups were performed using the Fisher’s exact test. Survival analysis was performed using Kaplan-Meier curves, and the p value was determined with the log-rank test, and hazard ratios (HRs) were calculated by Cox proportional hazards model. A two-sided p value of less than 0.05 was considered significant for all tests unless indicated otherwise. Univariable was used to study the association between different variables and PFS, and the results are presented as HRs and their 95% confidence intervals (CIs). All analyses were performed with R 3.4.0.

## Results

### Patient Characteristics

In this study, 17 patients were finally enrolled through strict screening, the clinical characteristics of these patients were summarized in **Table 1**. This study cohort includes ten patients with LCNEC and seven with LCC, which topical pathological images were shown in **Supplementary Figure S1**. The LCC and LCNEC cohorts were similar in their baseline characteristics. The median age of diagnosis of the study cohort was 62 years (LCC, 64 years; LCNEC, 62 years). There was an overall enrichment of male patients, with only one female patient in the LCC cohort. No clear association with smoking history was seen in either of the histological subtype. Stage IV patients accounted for about 70.6% of the patients included in the analysis, and stage III patients accounted for 29.4%. The correlation was analyzed between TNM stage and survival separately, and no correlation were found (p=0.420, HR 0.56, 95% CI, 0.13~2.34) (**Supplementary Figure S2**).

**Table 1:** Clinical characteristics of the patients enrolled in this study (n = 17).

Characteristics	Total (n = 17), n (%)	LCC (n = 7), n (%)	LCNEC (n = 10), n (%)
Age, years (median)	62 (44-80)	64 (55-80)	62 (44-73)
≤65	11 (64.7%)	4 (57.1%)	7 (70.0%)
>65	6 (35.3%)	3 (42.9%)	3 (30.0%)
Sex			
Female	1 (5.9%)	1 (14.3%)	0 (0%)
Male	16 (94.1%)	6 (85.7%)	10 (100%)
Smoking history			
Never	8 (47.1%)	4 (57.1%)	4 (40.0%)
Ever	9 (52.9%)	3 (42.9%)	6 (60.0%)
TNM Stage			
III	5 (29.4%)	2 (28.6%)	3 (30.0%)
IV	12 (70.6%)	5 (71.4%)	7 (70.0%)
ECOG PS			
0	4 (23.5%)	1 (14.3%)	3 (30.0%)
1	13 (76.5%)	6 (85.7%)	7 (70.0%)

LCC, large cell carcinoma; LCNEC, large cell neuroendocrine carcinoma.

**Figure 1:**
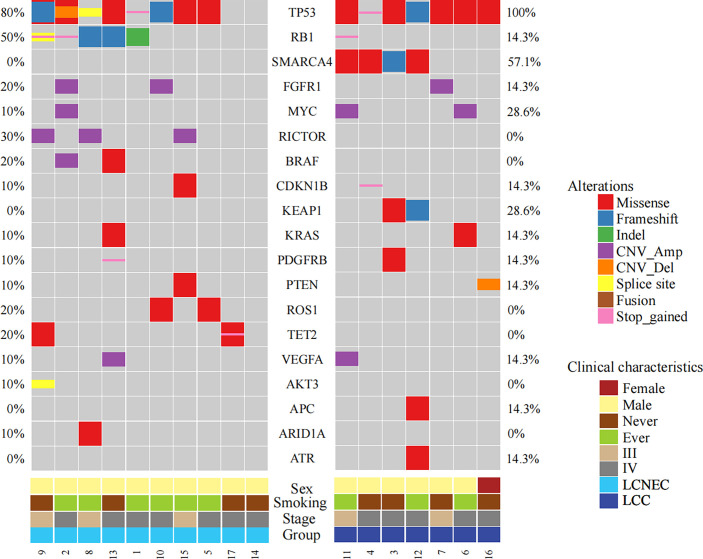
Mutational profiles comparing LCC and LCNEC. The top frequently mutated genes in the study cohort were shown with mutation frequencies in each subgroup indicated.

Considering only the overlapping genes in the two types of panels used in the study (Methods), the top 20 altered genes in the study cohort were shown in **Figure 1**, with LCC and LCNEC showed distinct mutation profiles. *TP53* was the most highly altered gene in the two histologic subgroups. We observed an enrichment of *TP53*/*RB1* co-mutations in a subset of LCNEC patients as previously reported (4, 6). In contrast, the co-occurrence of *TP53* and *RB1* mutations were rare in patients with classic LCC. We also noted that mutations in *SMARCA4* and *KEAP1* were exclusively detected in patients with classic LCC, whereas *RICTOR*, *BRAF*, *ROS1* and *TET2* mutations were only detected in those with LCNEC. *BRAF* alterations include gene copy number variation (CNV) (patient #2) and p.G460R (patient #13). *ROS1* alterations include p.F1828L (patient #5) and p.Y1696C (patient #10).

Several potentially targetable mutations were also identified, including mutations in *KRAS* and *RET*. *KRAS* mutations were detected in one LCC patient (p.G12V) and one LCNEC patient (p.G12C). We also identified a *KIF5B*-*RET* fusion gene in one case of LCNEC patient (patient #14).

### Association Analysis of Survival

**Figure 2:**
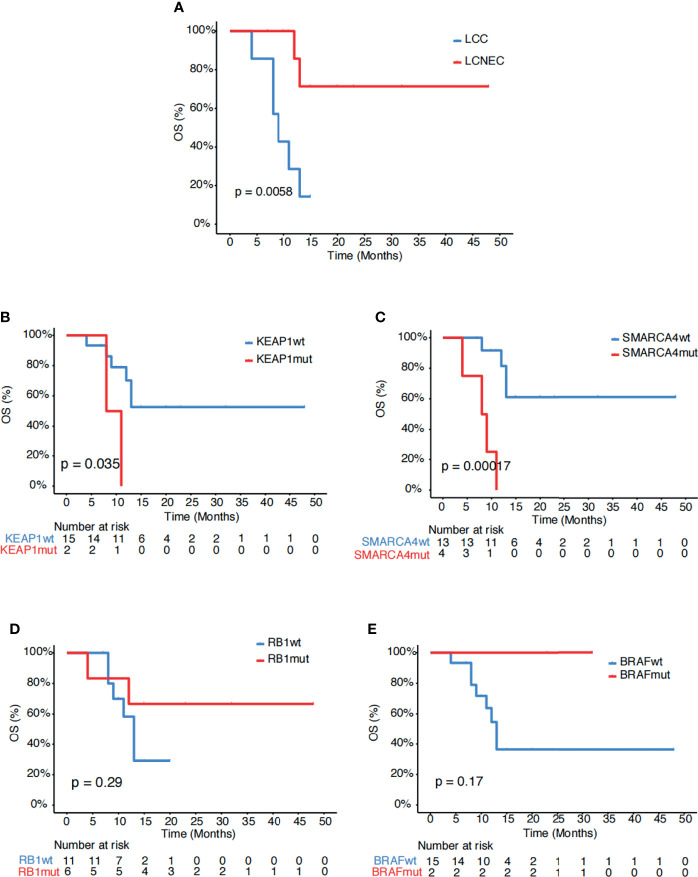
Associations of survival with genetic mutations. **(A)** Kaplan-Meier estimates of OS comparing LCC and LCNEC. **(B–E)** Kaplan-Meier estimates of OS comparing the subgroups with and without mutations in **(B)** *KEAP1*, **(C)** *SMARCA4*, **(D)** *RB1* and **(E)** *BRAF*.

Next, we explored for biomarkers that might be associated with survival in our study cohort. Comparing the two subgroups of patients, those with LCNEC showed longer survival than those with LCC (p=0.006, **Figure 2A**). Mutational analysis revealed several genes might be associated with differential survival outcome between the two groups. Mutations in *KEAP1* (p=0.035, **Figure 2B**) and *SMARCA4* (p<0.001, **Figure 2C**), which were detected only in the LCC group, were associated with poor survival. On the other hand, mutations in *RB1* (p=0.29, **Figure 2D**) and *BRAF* (p=0.17, **Figure 2E**), which were enriched in the LCNEC group, showed trends of increased survival.

**Figure 3:**
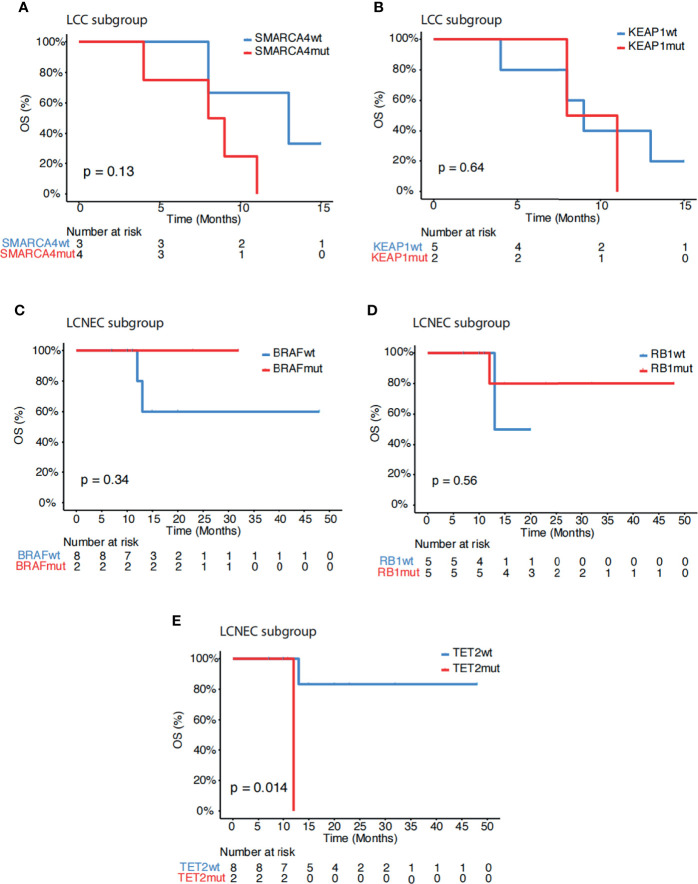
Subgroup association analysis of survival. **(A, B)** Kaplan-Meier estimates of OS comparing LCC patients with and without mutations in **(A)** *SMARCA4* and **(B)** *KEAP1*. **(C–E)** Kaplan-Meier estimates of OS comparing LCNEC patients with and without mutations in **(C)** *BRAF*, **(D)** *RB1* and **(E)** *TET2*.

Further analysis showed that *SMARCA4* mutations (p=0.13, **Figure 3A**), but not *KEAP1* mutations (p=0.64, **Figure 3B**) *remained* associated with poor survival within the LCC subgroup. Within the LCNEC subgroup, *BRAF* mutations (p=0.34, **Figure 3C**), but not *RB1* mutations (p=0.56, **Figure 3D**), still showed a trend of increased survival. In addition, mutations in *TET2* were associated with poor outcome in the LCNEC subgroup (p=0.014, **Figure 3E**). No other genetic alterations showed associations with survival in this study cohort. Systemic treatment received in our study is depicted in **Supplementary Table S1**.

## Discussion

In this study, we examined the mutational profiles of LCC and LCNEC patients and conducted association analysis of survival for the identification of potential biomarkers of diagnostic and prognostic value. LCC and LCNEC are two subtypes of lung cancer of heterogenous nature and limited treatment options. Due to relatively difficult diagnosis, detailed pathological diagnosis was finally confirmed by experienced pathologists in this study. The number of cases included in the study was relatively small but ensured the authenticity of subsequent genetic testing results. In this study, we identified several targetable mutations in both LCC and LCNEC, including *KRAS* G12 mutations and a *RET* fusion gene. This finding suggests that genetic profiling might be necessary in such patients as it might provide more therapeutic options which is available using, tumor DNA or cell-free DNA (cfDNA) to monitor these mutations (14).

While the number of patients in our study was not large, all patients received platinum-based first-line therapy in both LCNEC and LCC. In additional, our results are similar to the observations in reported study (15), 52.9% of patients received second-line chemotherapy, which was also platinum-based. In contrast to another previous report (2), we found that LCNEC patients had better survival compared to patients with classic LCC, which might be associated with the enrichment of *RB1* and *BRAF* mutations in the LCNEC group and the enrichment of *SMARCA4* and *KEAP1* mutations in the LCC group. Previous studies have shown that *KEAP1* mutations were known to be a typical characteristic of NSCLC-like LCNEC, and more likely to be resistant to chemotherapy and EGFR-TKI drugs (6, 16). LCNEC samples suggest that LCNEC can be subdivided into different subtypes, partly clustering with SCLC. By comparative analysis of 69 LCNECs and 110 SCLCs, George et al. have found that despite their mutational patterns, LCNECs with KEAP1 mutations exhibit a neuroendocrine profile with closest similarity to SCLC tumors (17). Rekhtman et al. also showed that significantly higher rate of *KEAP1* alterations (33%) was found in SCLC-like LCNEC, differed from SCLC with 5% of *KEAP1* alterations (4). The incidence of *KEAP1* mutation is not high, especially in the Chinese lung cancer patients, with the reported incidence of about 20%. *KEAP1* mutation was only found in LCC patients, but not in LCNEC in this study. Possible reasons are the small sample, and the neuroendocrine profile with closest similarity to SCLC in the LCNEC subtype. This is consistent with the poor survival of LCC patients with *KEAP1* mutations shown in our data.

Within the LCC subgroup, the presence of *SMARCA4* mutations remained associated with poor survival outcome. *SMARCA4*, encoding the BRG1 protein, participates in the chromatin remodeling process and DNA repair and is frequently mutated in lung cancer (18, 19). Low expression of SMARCA4 has been associated with worse prognosis in patients with non-small-cell lung cancer (18). Co-mutations occurred more frequently with *SMARCA*4 mutations than with *SMARCA4* wild-type tumors. In our data, four cases were co-mutated with *TP53* and two cases were co-mutated with *KEAP1*, and also the co-mutated patients showed shorter survival time. Recent studies have found that treatment with immune checkpoint inhibitors is associated with improved outcomes in patients with *SMARCA4* mutated, suggesting that *SMARCA*4 mutated lung cancer may be more sensitive to immunotherapy. *SMARCA4* mutation detection may be required in our future studies to further explore its correlation with immunotherapy (20). Within the LCNEC group, *BRAF* mutations showed a trend of increased survival. In addition, *TET2* mutations might serve as a negative prognostic marker in the LCNEC subgroup.

## Conclusion

In summary, we reported potentially targetable mutations in both LCC and LCNEC, and identified several novel genetic alterations that might serve for diagnostic and prognostic purposes. Given the relative uncommon nature of LCC and LCNEC and therefore the limited sample size in our cohort, the diagnostic and prognostic role of the reported genes would require further investigations in larger-sample cohorts.

## Data Availability Statement

The datasets presented in this study can be found in online repositories. The names of the repository/repositories and accession number(s) can be found below: Genome Variation Map, National Genomics Data Center, GVM000288.

## Ethics Statement

Written informed consent was obtained from the individual(s) for the publication of any potentially identifiable images or data included in this article.

## Author Contributions

XD contributed to study design and implementation. YC was the principal investigator and wrote the report. Sample collection was done by XC, DW, and YC. GX advised on the trial. Analysis and interpretation of data were done by YC, LC, MX, and LS. Writing, review, and/or revision of the manuscript were done by YC. All authors contributed to the interpretation of data. All authors contributed to the article and approved the submitted version.

## Conflict of Interest

Author DW was employed by Nanjing Geneseeq Technology Inc.

The remaining authors declare that the research was conducted in the absence of any commercial or financial relationships that could be construed as a potential conflict of interest.

## Publisher’s Note

All claims expressed in this article are solely those of the authors and do not necessarily represent those of their affiliated organizations, or those of the publisher, the editors and the reviewers. Any product that may be evaluated in this article, or claim that may be made by its manufacturer, is not guaranteed or endorsed by the publisher.

## Supplementary Material

Supplementary Figure S1 Topical pathological images for both LCNEC and LCC of the cases in this study. Pathological examination of primary lesion biopsy. The LCC H&E staining images of patient #12 **(A)** and LCNEC H&E staining images of patient #11 **(B)** were shown. The magnificence of the images were 100X (Left) and 400X (Right) for both two patients, respectively

Supplementary Figure S2 Kaplan-Meier curve of OS in strata of different disease stages.
