# Supplementary figures and images for: Molecular Characterization and Prognostication of Large Cell Neuroendocrine Carcinoma and Large Cell Carcinoma
Source: Front Oncol. 2022 Jan 14;11:664397. doi: 10.3389/fonc.2021.664397 (PMC8796852; doi:10.3389/fonc.2021.664397)

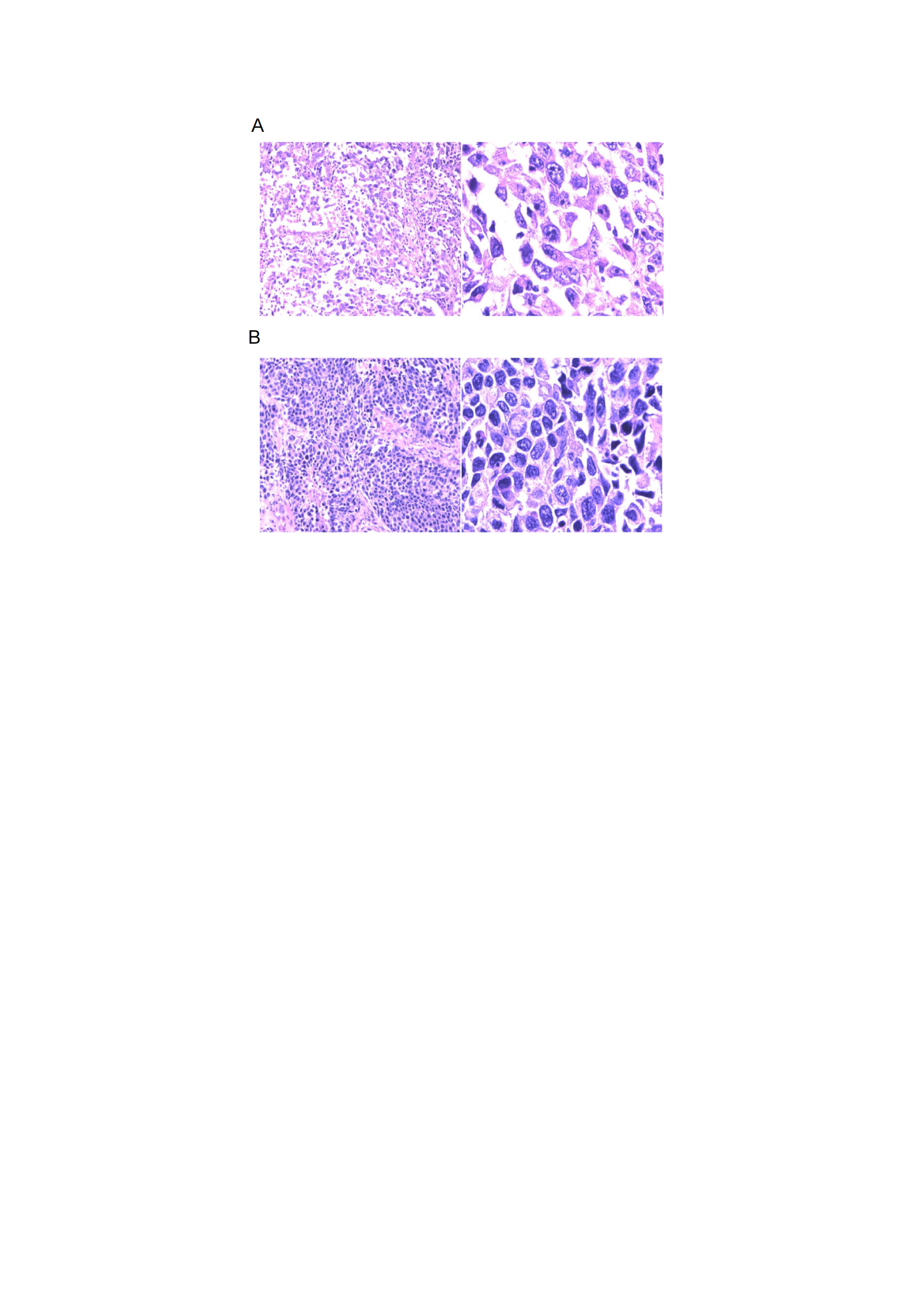

Supplement: Supplementary Figure S1 — Topical pathological images for both LCNEC and LCC of the cases in this study. Pathological examination of primary lesion biopsy. The LCC H&E staining images of patient #12 (A) and LCNEC H&E staining images of patient #11 (B) were shown. The magnificence of the images were 100X (Left) and 400X (Right) for both two patients, respectively [file Image_1.tif]

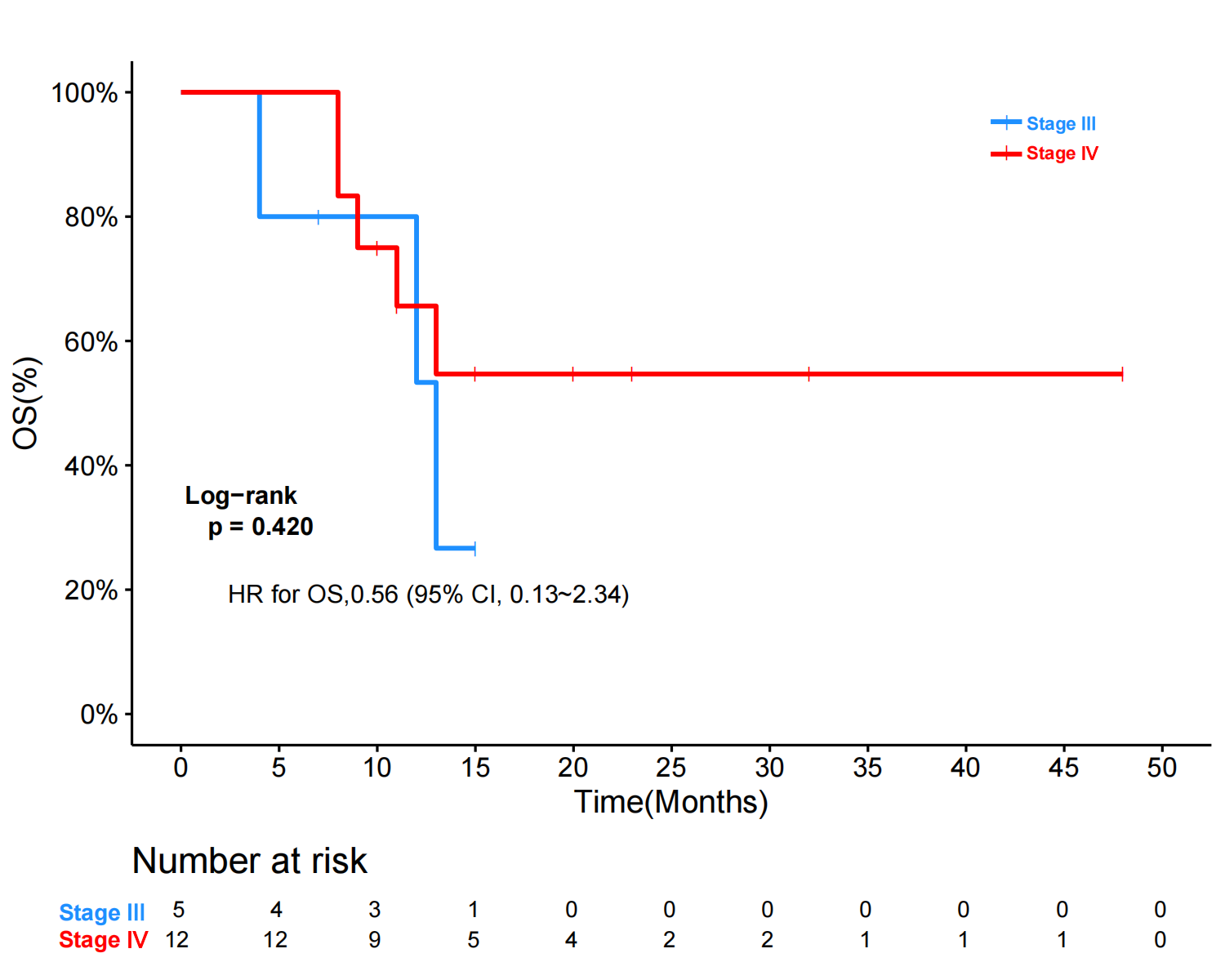

Supplement: Supplementary Figure S2 — Kaplan-Meier curve of OS in strata of different disease stages. [file Image_2.tif]
