# Supplementary material for: Molecular Characterization and Prognostication of Large Cell Neuroendocrine Carcinoma and Large Cell Carcinoma
Source: Front Oncol. 2022 Jan 14;11:664397. doi: 10.3389/fonc.2021.664397 (PMC8796852; doi:10.3389/fonc.2021.664397)
Supplement: Supplementary file 3 [file Table_1.docx]

**Table S1**. Systemic treatment received by patients of this study.

| Patient ID | Histological  Type | TNM  Stage | Survival-related mutation | 1^st^ line | 2^nd^ line | 3^rd^ line |
| --- | --- | --- | --- | --- | --- | --- |
| 1 | LCNEC | IV | *RB1* | EC | Camrelizumab  plus EC | Anlotinib plus EP |
| 2 | LCNEC | IV | *BRAF;*  *RB1* | Endostatin plus EP | Camrelizumab plus EP | - |
| 3 | LCC | IV | *SMARCA4;*  *KEAP1* | EP | BEV plus PC | - |
| 4 | LCC | IV | *SMARCA4* | PC | BEV plus PC | - |
| 5 | LCNEC | IV | - | EP | Traditional Chinese medicine | - |
| 6 | LCC | IV | - | BEV plus PC | - | - |
| 7 | LCC | IIIA | - | BEV plus PC | - | - |
| 8 | LCNEC | IIIB | *RB1* | EC | IC | - |
| 9 | LCNEC | IIIA | *TET2;*  *RB1* | IP | - | - |
| 10 | LCNEC | IV | - | EP | IC |  |
| 11 | LCC | IIIC | *SMARCA4;*  *RB1* | PC | - | - |
| 12 | LCC | IV | *SMARCA4;*  *KEAP1* | BEV plus PC | - | - |
| 13 | LCNEC | IV | *BRAF;*  *RB1* | EC | Camrelizumab plus EC | IP |
| 14 | LCNEC | IV | - | EC | - | - |
| 15 | LCNEC | IIIB | - | EC | - | - |
| 16 | LCC | IV | - | PC | Camrelizumab plus PC | - |
| 17 | LCNEC | IV | *TET2* | EP | IP | - |

LCC, large cell carcinoma; LCNEC, large cell neuroendocrine carcinoma; PC, Pemetrexed plus Carboplatin; EP, Etoposide plus Cisplatin; EC, Etoposide plus Carboplatin; IP, Irinotecan plus Cisplatin; IC, Irinotecan plus Carboplatin; BEV, bevacizumab.
